# Deciphering the impact of TERT/telomerase on immunosenescence and T cell revitalization

**DOI:** 10.3389/fimmu.2024.1465006

**Published:** 2024-09-23

**Authors:** Lingyi Huang, Mingfu Zhang, Ding Bai, Yi Qu

**Affiliations:** ^1^ Department of Orthodontics, West China College of Stomatology/State Key Laboratory of Oral Diseases, Sichuan University, Chengdu, China; ^2^ Key Laboratory of Birth Defects and Related Diseases of Women and Children (Ministry of Education), West China Second University Hospital, Sichuan University, Chengdu, China

**Keywords:** immunosenescence, telomerase reverse transcriptase (TERT), telomerase, CD28, T lymphocytes

## Abstract

Immunosenescence impacts both the innate and adaptive immune systems, predominantly affecting certain immune cell types. A notable manifestation of immunosenescence is the diminished efficacy of adaptive immunity. The excessive senescence of immune cells, particularly T cells, leads to marked immune deficiency, consequently escalating the risk of infections, tumors, and age-associated disorders. Lymphocytes, especially T cells, are subject to both replicative and premature senescence. Telomerase reverse transcriptase (TERT) and telomerase have multifaceted roles in regulating cellular behavior, possessing the ability to counteract both replicative and premature senescence in lymphocytes. This review encapsulates recent advancements in understanding immunosenescence, with a focus on T cell senescence, and the regulatory mechanisms involving TERT/telomerase. Additionally, it comprehensively discusses strategies aimed at inhibiting immunosenescence by augmenting TERT/telomerase activity.

## 1 Introduction

Immunosenescence refers to the progressive alterations within various facets of the immune system, culminating in deficiencies of both adaptive and innate immunity (1). Key immune alterations during immunosenescence encompass a decline in the population of circulating naïve and effective T cells, an expansion of memory T cells, and elevated levels of pro-inflammatory cytokines such as TNFα and IL-6 (2). These changes collectively heighten the risk of infections, tumors, and age-related ailments, including cardiovascular, neurodegenerative, and metabolic diseases (3–5).

Cells undergoing senescence are typified by halted cell cycles, diminished proliferation, altered morphology, and a reduced propensity for apoptosis (6). Cellular senescence bifurcates into telomere-dependent replication senescence and premature senescence (7). Telomeres, consisting of repetitive (TTAGGG)n sequences at chromosome termini, are pivotal for maintaining robust cell proliferation (8). The “end replication problem” refers to the gradual loss of approximately 200 base pairs per telomere in each cell division due to incomplete lagging strand synthesis during DNA replication (9). This progressive telomere attrition eventually triggers a critical chromosomal length threshold, ushering cells into a state termed “replicative senescence” (10). On the other hand, premature senescence (PS) represents a telomere-independent, stress-induced senescence, commonly activated by damaging agents like oxidative stress (11). Oxidative stress can inflict various DNA damages, including single-strand breaks (SSBs) and double-strand breaks (DSBs), precipitating non-telomeric DNA damage responses and senescence (12, 13). Lymphocytes, particularly T cells, may enter replicative senescence following numerous divisions and proliferation cycles due to natural senescence or persistent antigen exposure. Conversely, lymphocytes may succumb to premature senescence resulted from oxidative damage under detrimental conditions, such as infection and stress.

Immunosenescence is associated with a chronic, low-grade inflammatory state termed “inflammaging” (14). Studies indicate that T cells are predominantly responsible for this age-related inflammatory milieu (15, 16). Certain cytokines secreted by senescent T cells can promote the senescence of nearby and distant cells (15). Granzyme K (GZMK), mainly released by T cells, intensifies the senescence-associated secretory phenotype (SASP) of senescent cells, further amplifying pro-inflammatory factor production (16). GZMK is a member of the granzyme family which induces proinflammatory phenotypes (17). Mogilenko et al. found that, in mouse 3T3 fibroblasts, exogenously added GZMK dramatically boosted IFNg induced secretion of IL-6 and CCL5, factors of inflammation increased in aging, they also found that GZMK itself could significantly increase SASP components such as IL-6, CCL2, and CXCL1 in fibroblasts. This indicates that GZMK, alone or in combination with IFNg, has the potential to exacerbate the SASP and suggests it may be an important regulator of inflammatory processes in aging (16). Senescent CD4+T cells develop cytotoxic traits and emit harmful substances that inflict direct tissue damage (18). As immune cells senesce, irreparably damaged tissue cells persist and accumulate, exacerbating the inflammatory response (19). Hence, senescent T lymphocytes are primary contributors to the manifestations of immunosenescence. Accordingly, this review concentrates on T cell senescence and its regulation through telomerase reverse transcriptase (TERT) and telomerase.

Senescent T cells are characterized by telomere shortening, loss of CD28 expression, and cell cycle arrest. Besides, high expression of CD57, Tim-3, killer cell lectin like receptor subfamily G member 1 (KLRG-1) are regarded to be associated with T cell senescence (1). T cell senescence is easy to confuse with other dysfunctional T cell states such as exhaustion and anergy. T cell exhaustion is described as effector T cells with attenuated effector function and cytokine expression, and decreased ability of reactivation. Typically, B7-H1/PD-1 signaling pathway is regarded to mediate CD8+ T cell functional exhaustion, and PD-1 is proposed to be a marker for exhausted T cells. Notably, exhausted T cells may also highly express”inhibitory”receptors such as CD244, CD160, CTLA-4, BTLA, LAG-3 and Tim-3 (20). T cell anergy is commonly described as the hyporesponsive state with incomplete activation and decreased IL-2 production, as well as cell cycle arrest at the G1/S phase. It is generally regarded that T cells which are presented antigen along with low CD28 co-stimulation and/or high co-inhibition lead to anergic phenotypes (20). T-cell exhaustion and anergy share some overlapping molecular hallmarks and functional features with senescence; However, they have unique developmental signatures and specific regulatory mechanisms respectively, which has been thoroughly discussed and can be referred in the previously published reviews (1, 20).

## 2 Canonical and non-canonical roles of TERT/telomerase

TERT/telomerase functions in both canonical and non-canonical capacities to modulate cellular behavior (21). Its primary and canonical role involves countering replicative senescence (22). Telomerase composes TERT, the telomerase RNA component (TERC), and associated proteins (23). It primarily functions through its crucial catalytic subunit, TERT. TERT exhibits reverse transcriptase activity, facilitating the synthesis of linear chromosomal end (TTAGGG)n sequences and preserving telomere length and genomic integrity, thereby circumventing replicative senescence (24).

Beyond its canonical role, TERT/telomerase orchestrates a spectrum of non-canonical functions. These include the modulation of non-telomeric DNA damage responses, enhancement of cellular growth and proliferation, acceleration of cell cycle progression, and preservation of mitochondrial integrity under oxidative stress (25). TERT within the nucleus can safeguard cells from apoptosis following DNA damage, irrespective of its telomere-maintenance function (26). It also plays pivotal roles in shaping chromatin structure and regulating gene transcription (27–29) and has been implicated in generating siRNAs through a Dicer-dependent mechanism to modulate gene expression (30). Moreover, TERT within the cytoplasm is instrumental in protecting mitochondria from oxidative stress-induced mtDNA damage and in maintaining mitochondrial integrity (31). Collectively, these non-canonical actions provide comprehensive cellular protection, potentially aiding in the mitigation of premature senescence.

There is substantial evidence supporting TERT/telomerase’s regulatory influence on immune-related gene expression, epitomizing its non-canonical utility (32). TERT modulates gene expression by influencing chromatin architecture and/or interacting with transcription or chromatin-altering factors (33, 34). Notably, TERT associates with transcription factors like NF-κB and β-catenin, vital for cell proliferation and initiating immune responses (35). Within lymphocytes, TNF-α induces the nuclear translocation of TERT, a process facilitated by the PI3K-Akt-NF-κB signaling pathway (36). Once in the nucleus, TERT binds to the NF-κB p65 subunit, localizes to specific NF-κB promoter sites, and stimulates the expression of NF-κB-dependent genes, including IL-6 and TNF-α (33). TERT also collaborates with β-catenin as a co-activator within the β-catenin transcriptional complex, thereby influencing gene expression and cellular dynamics (33). Furthermore, the Wnt/β-catenin pathway, in which TERT is implicated, is known to regulate several immunological processes, such as sustaining regular T cell development, maintaining memory CD8+T cells, and directing CD4+Th2 differentiation (37, 38). These insights underscore TERT/telomerase’s critical role in regulating immune functions and immunosenescence by regulating key immune-related transcription factors.

It is worth noting that in addition to TERT, the production of immune regulatory factors is also influenced by multiple issues. For example, Parish et al. observed that sustained CD28 expression greatly reduced the secretion of IL-6 and TNF-α, while increased expression of IFN-γ and IL-2, thus was involved in the maintenance of a”more youthful”cytokine secretion pattern (39). Under circumstance of immunosenescence, although diminished TERT might attenuate the expression of IL-6 and TNF-α, reduced CD28 might facilitate their expression, hence the comprehensive effect is reflected as elevated levels of IL-6 and TNF-α.

## 3 TERT/telomerase regulating immunosenescence especially T cell senescence

Immunosenescence impairs both the innate and adaptive immune systems, particularly affecting certain immune cell types (40). A notable aspect of immunosenescence is the compromised adaptive immunity (41). Excessive senescence of immune cells, especially T cells, results in marked immune deficiency (42). While most somatic cells lack TERT expression and telomerase activity (43), T cells inherently express TERT and demonstrate telomerase activity, tightly regulated throughout T cell development and activation (44). Antigen stimulation triggers rapid TERT activation in both CD8+ and CD4+ T cells. Although TERT activity markedly increases after initial stimulation, its expression dwindles in subsequent stimulation cycles. TERT expression becomes nearly undetectable as cells approach senescence (45, 46), signifying stringent regulation of TERT expression and telomerase activity in T cells.

Typically, in an immune response, a naïve cell proliferates into a million activated effector cell clones through 15-20 cell divisions (47). Persistent cell division induces telomere attrition and replicative senescence. Memory CD8+ T cells, compared to their naive counterparts, and memory CD4+ T cells, in relation to naive CD4+ T lymphocytes, exhibit shorter telomeres (48, 49). This illustrates that T lymphocyte differentiation from naïve to memory cells involves telomere depletion and shortening. During persistent T cell activation by certain pathogens, like HIV, diminished TERT activity contributes significantly to telomere shortening and the gradual decline of antigen-specific T cell replication capacity (46).

Upregulation of telomerase requires T cell stimulation through the antigen receptor (TCR) and costimulatory receptors, among the costimulatory receptors, CD28 is the most important one (50). As naïve T cells differentiate into memory T cells, a reduction in TERT activity usually coincides with diminished CD28 co-stimulatory molecule presence (51). Similarly, the frequency of CD28+ cells decrease with senescence (52). Senescent CD28- T cells, characterized by low telomerase activity and shorter telomeres (53), reveal that excessive antigen exposure leads to reduced CD28 expression in T cells, coinciding with decreased telomerase activity and an increased number of senescent T cells, hence underscoring the necessity of CD28 expression for telomerase (54, 55). Inhibiting CD28 binding to its receptors B7-1 and B7-2 on APCs significantly lowers telomerase activity during antigen stimulation, underscoring CD28’s role in telomerase regulation (56). After *in vitro* activation, CD28+ T cells sorted from a broader T cell population display heightened telomerase activity (57). Additionally, CD28− T cells have shorter telomeres than CD28+ cells from the same donor (58). A study using retroviral vectors to increase CD28 expression in T cells revealed that, compared to control T cells transfected with an empty vector, T cells with heightened CD28 expression exhibited augmented telomerase activity and telomere elongation (39). Collectively, these studies robustly suggest that CD28 signaling is crucial in promoting TERT expression and telomerase activity, thus preserving telomere length in T cells. Intriguingly, TNFα, typically released by senescent non-immune somatic cells or activated lymphocytes, has been observed to suppress CD28 gene transcription, indicating that senescent or inflamed cells contribute to T cell senescence by secreting this cytokine (59). Furthermore, studies indicate that in senescent or persistently activated macrophages, cyclooxygenase-2 activity rises, enhancing the catalytic generation of prostaglandin E2 (PGE2). Under PGE2 influence, T cells exhibit a senescent phenotype characterized by decreased CD28 protein expression, telomerase activity, and T cell proliferation, substantially impairing their tumor surveillance and elimination capabilities (60). There is no direct evidence to show how PGE2 regulates the expression of CD28 and TERT in T cells. Apart from transcriptional regulation of CD28 gene expression, there exists post-translation modification mechanisms of CD28 protein. Parish et al. found that CTLA-4 message increased with culture age of human CD8 T lymphocytes, and CTLA-4 enhanced loss of CD28 protein through a post-translational modification mechanism, probably accelerating its degradation (39). Furthermore, Sajiki found that PGE2 exerted its immunosuppressive effects through inducing CTLA-4 expression in T cells in a paratuberculosis animal model (61). Taken together, these findings suggest that PGE2 might induce CTLA-4 expression in T cells, which in turn accelerate CD28 protein loss, finally attenuate TERT expression and telomerase activation.

**Figure 1:**
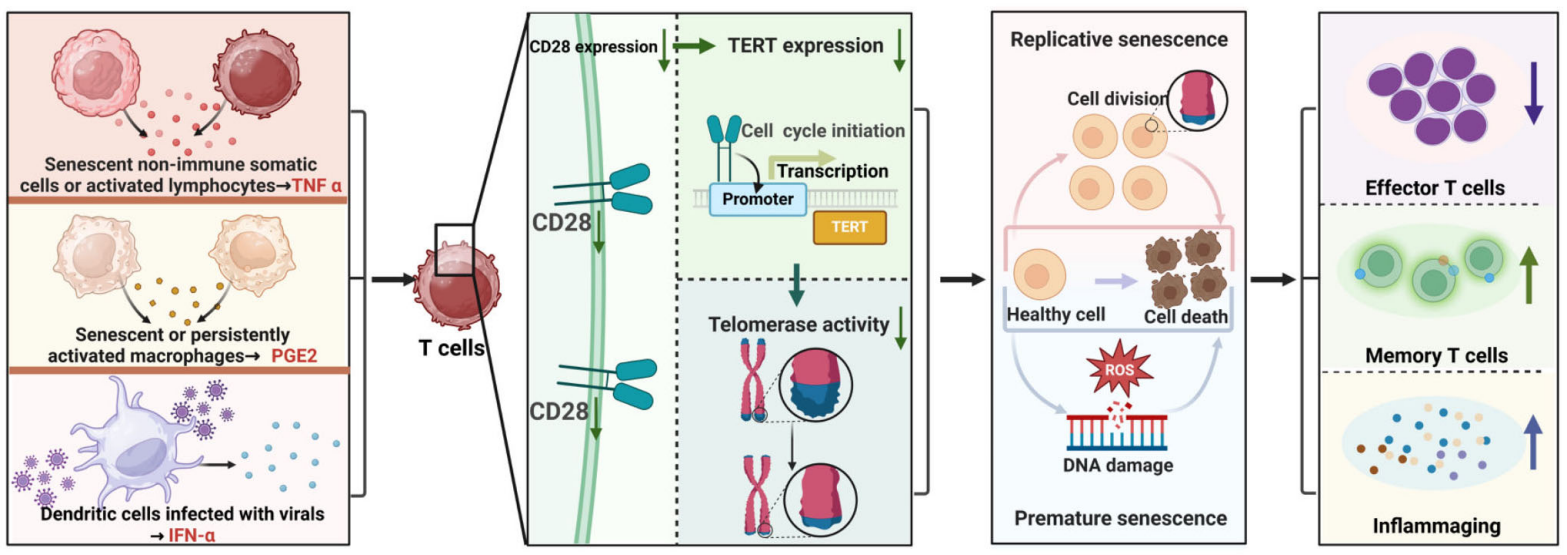
Primary Mechanism of TERT/Telomerase Regulation in T Cell Immunosenescence. Some factors such as TNFα, PGE2 and IFN-α released by senescent, activated or infected non-immune somatic cells or immune cells down-regulate CD28 protein expression, attenuate TERT expression and telomerase activation in T cells, thereby weakening the resistance of TERT and telomerase to premature and replicative senescence of T cells, resulting in reduce of effector T cells, increase of memory T cells, and enhancement of inflammaging.

Specifically, T cell senescence can be triggered by viral infections such as human immunodeficiency virus (HIV), cytomegalovirus (CMV), hepatitis B/C/D virus (HBV/HCV/HDV), human herpesvirus 8 (HHV-8), and so on (62). Studies have documented alterations in T cell telomerase activity following exposure to these viruses. For instance, in HIV-1 viremic individuals, virus-specific CD8+ T cells from spontaneously controlling subjects demonstrate enhanced telomerase activity and extended telomere lengths compared to virus-specific CD8+ T cells from patients experiencing HIV-1 progression (63). Additionally, a rise in CMV IgG levels in CMV serum-positive individuals correlates with reduced telomerase activity (64). Moreover, patients with HBV/HCV exhibit lower hTERT mRNA levels in PBMCs compared to healthy individuals (65). Similarly, individuals initially infected with EB virus show high telomerase activity and lengthy telomeres in virus-specific T cells; however, these specific T cells later display diminished telomerase activity and shortened telomere lengths (66). Mechanistically, chronic viral infection can prompt dendritic cells to release IFN-α, which can inhibit both the transcription and translation of TERT, resulting in decreased telomerase activity in T cells (67). **Figure 1** illustrates the primary mechanism through which TERT/telomerase regulates T-cell immunosenescence.

There exists distinct paths other than TERT/telomerase to regulate T cell senescence. It has been reported that a mutation in tripeptidyl peptidase II (TPP2) or in phosphoinositide 3-kinase (PI3K), leads to TPP2 deficiency or activated PI3K, finally results in premature senescence of T cells and immunodeficiencies (68–70). Activated PI3 kinase delta syndrome (APDS) has received extensive research. APDS patients carry heterozygous gain-of-function mutations in PIK3CD (APDS1) or loss-of-function mutations in PIK3R1 (APDS2), resulting in enhanced PI3K and activated downstream Akt/mTOR signaling. Sustained hyperactivity of Akt/mTOR drives T cells to differentiate into terminal effector T cells, with accumulation of senescent CD57+CD8+ T cells which proliferate poorly. However, there was much less pronounced telomere loss during the transition from the naive state to the CD57+ differentiation state (70). Previous studies have revealed that hyperactive Akt/mTOR signaling led to telomerase activation (54, 70, 71), which might be the reason contributing to the limited telomere shortening of senescent T cells in APDS patients.

Apart from the conventional view that T lymphocytes delay senescence by activating telomerase, another study suggests that some T cells (mainly naïve and central memory cells) acquire telomere vesicles from antigen-presenting cells (APCs) independently of telomerase activity, thereby extending their telomeres (72). These telomeric vesicles contain the Rad51 recombination factor, enabling telomere fusion to the ends of T-cell chromosomes and resulting in an average extension of approximately 3000 base pairs. T cells that receive telomeres are safeguarded against senescence prior to the onset of clonal division.

Analogous to T cells, B cells experience age-related telomere shortening, albeit at a slower pace than T cells (73). Telomerase is not present in resting B cells but is swiftly activated upon antigen stimulation (74). Notably, there is no significant telomere length discrepancy between naïve and memory B cells, nor does telomere length diminish during the transition from naïve to memory B cells (75). It was hypothesized that the preservation of telomeres in B cells depends primarily on the telomerase expression as it does not occur in telomerase deficient animals (76). Moreover, moderate levels of telomerase were detected in terminally differentiated B cells (77), further confirming this hypothesis.

Similar to adaptive immunity cells, innate immunity cells undergo telomere shortening during senescence (78). While myeloid progenitor cells express telomerase, mature granulocytes, monocytes, and mast cells do not, and they do not undergo cell division. Therefore, the telomere lengths in these cells reflect the telomere attrition in myeloid progenitor cells (78). Contrasting with mature granulocytes, monocytes, and mast cells, NK cells can proliferate following antigen stimulation. Therefore, telomerase activity in mature NK cells diminishes with senescence, leading to significantly shorter telomeres in mature NK cells compared to their immature counterparts (79).

## 4 T cell-related phenotypes/pathogenesis in telomere biology disorders

Germline mutations of TERT or other telomerase-relevant genes are associated with a set of tissue-failure diseases characterized by very short telomeres. These diseases are known as telomere biology disorders (TBDs). The classic TBD was initially defined as dyskeratosis congenita (DC), which is a systemic disease characterized by abnormal skin pigmentation, dystrophic nails, bone marrow failure, as well as predisposition to specific cancers. DC is genetically heterogeneous and patients have mutations in genes that encode components of the telomerase complex (TERC, TERT, PARN, DKC1, RTEL1, NOP10 and NHP2), and telomere shelterin complex (TINF2), both important for telomere maintenance (23).

Knudson et al. have described 10 DC patients with mutations in the gene encoding human telomerase RNA (TERC), resulting in telomere shortening. In studying the immunologic consequences of TERC mutations, T cells were found to overexpress senescent markers, including CD57 and Fas receptor, and were moderately reduced in cell number. Furthermore, DC lymphocytes displayed a markedly reduced proliferative capacity and increased apoptotic rate. The observed immunodeficiency in DC is probably due to the replicative failure and premature senescence of lymphocytes, supporting a role of telomerase in regulating immune homeostasis (80).

Zeng et al. summarized the clinical data of two juvenile patients with DC. Gene sequencing showed Patient 1 had a compound heterozygous mutation (c.204G > T) in PARN and Patient 2 had a novel mutation in DKC1 (c.1051A > G). Short telomere lengths, increased CD57 expression, and an expansion of CD8 effector memory T cells re-expressing CD45RA (TEMRA) were found in both patients. They concluded that unique immunologic abnormalities, CD8 T-cell senescence, and shortened telomere together as a hallmark occur in young DC patients (48).

Kirwan et al. transduced primary T lymphocytes and B lymphocyte lines established from DC patients carrying TERC and DKC1 mutations with wild type TERC-bearing lentiviral vectors. They found that transduction with exogenous TERC alone was capable of increasing telomerase activity in mutant T lymphocytes and B lymphocyte lines and improved their survival. Telomeres in TERC-treated lines were longer than that in the untreated cultures, indicating that extending the telomere length of lymphocytes in DC patients can reverse immune deficiency (81).

Patients with TBDs are predisposed to developing cancer, which was regarded to be resulted from chromosome instability in neoplastic cells previously. Schratz et al. carried out a detailed study of the immune status of patients with TBDs, which revealed a striking T cell immunodeficiency at the time of cancer diagnosis. A similar immunodeficiency that impaired tumor surveillance was also found in mice with short telomere, indicating that TBDs patients’ predisposition to solid cancers is due to T cell deficiency rather than autonomous defects in the neoplastic cells themselves (82).

Patients with inherited CARMIL2 or CD28 deficiency have defective T cell CD28 signaling, characterized by dysfunction in naive T-cell activation, proliferation, differentiation, and effector function (83). Recently, Zhu et al. reported a 9-year-old female patient with a novel pathogenic variant in CARMIL2 (c.2063C > G) who presented with various symptoms of primary immunodeficiencies. The missense mutation leading to insufficient CARMIL2 protein expression, reduced absolute T-cell and NK cell counts, and defective maturation of T cells and B cells (84). There is no direct report in the existing literature on the role of TERT in CARMIL2 or CD28 deficiency. Given the importance of CD28 in TERT/telomerase induction, we speculate that CARMIL2 or CD28 deficiency might dampen TERT expression and telomerase activation, thus facilitate T cell senescence.

## 5 Targeting TERT/telomerase to mitigate immunosenescence

*In vitro* studies involving cultured cells have demonstrated that enhanced expression of TERT/Telomerase can thwart cell senescence and foster cell proliferation (85, 86). Alongside endothelial cells and fibroblasts (87, 88), lymphocytes are also amenable to TERT/telomerase manipulation. Elevating TERT levels significantly extends the replication longevity of antigen-specific T cell lines and clones, preserving their inherent T cell attributes (89). Consequently, T cells immortalized via TERT overexpression are anticipated to serve as potent instruments for adoptive transfer immunotherapy. Experiments utilizing mouse tumor models reveal that TERT-augmented tumor-active T cells retain robust antitumor responses. The human melanoma cell line melAKR, modified to express influenza virus epitopes via retrovirus transfection, formed ectopic tumors upon mouse implantation. An influenza virus-specific human CTL clone, modified with TERT, was employed in adoptive transfer therapy, effectively curbing the growth of melAKR Flu tumors in mice and prompting their regression (90). This suggests that TERT-modified T cells preserve antigen-specific and comprehensive immune responses *in vivo*, hinting at their potential to combat tumors through adoptive immunotherapy. Moreover, TERT overexpression shields T cells from apoptosis. Compared to their unaltered counterparts, TERT-enhanced T cells exhibit higher levels of the anti-apoptotic protein Bcl2, reduced caspase-3 activity, and bolstered resilience against oxidative stress stemming from telomere DNA damage (91, 92). Thus, TERT overexpression not only safeguards T cells from replicative senescence but also fortifies their resistance to apoptosis, enhancing T cell survival. The underlying mechanism of immune system deterioration in AIDS is linked to the premature senescence of HIV-1 specific T cells and the diminished expression of related effector molecules, such as granzyme and perforin. This premature senescence and the consequent decline in immune responsiveness of HIV-1 specific T cells can be mitigated by transfecting T cells with TERT expression vectors (93). A recent investigation revealed that artificially inducing TERT expression in NK cells amplifies their activation, proliferation, and longevity, suggesting that telomerase replenishment in NK cells might be a viable strategy for NK cell-based cancer therapies (79).

In addition to genetic manipulation of the TERT gene, pharmacological strategies can also activate TERT. Following exposure to the plant-derived telomerase activator TAT2 in cell culture, CD8+ T cells from HIV-infected individuals demonstrated increased telomerase activity, delayed telomere attrition, enhanced proliferation potential, and a boosted production of functional cytokines and chemokines, contributing to HIV resistance (94). The introduction of specific telomerase inhibitors alongside TAT2 into the culture medium negates these effects entirely (94). A more nuanced investigation into human T lymphocytes revealed that a particular dosage of atorvastatin temporarily boosts telomerase activity, with a more pronounced effect in CD4+T cells compared to the CD8+ subset (95). Moreover, the upsurge in telomerase activity in CD4+T cells coincides with increased cell proliferation and a slowdown in inflammatory responses within arterial vessel walls, thereby decelerating atherosclerosis progression (95). Another study highlighted that IL-15 significantly promotes TERT enhancement in NK, NKT-like cells, and CD8 T cells, marking it as a valuable asset for adoptive cell therapies. Further analysis using signaling pathway inhibitors pinpointed that the JAK/STAT and PI3K/AKT pathways are crucial for IL-15’s role in augmenting TERT expression in NK and NKT cells, while CD8 T cell TERT expression is mediated through the JAK-STAT, PI3K-AKT, and Ras-MAPK pathways (96). A recent double-blind randomized controlled trial evaluated the impact of TA-65, a telomerase activator derived from Astragalus membranaceus, on mitigating immune cell senescence post-myocardial infarction (MI) (97). MI is known to expedite immunosenescence, characterized by lymphopenia, proliferation of terminally differentiated CD8+ T lymphocytes (CD8+ TEMRA), and inflammation. Ninety MI patients aged 65 and above were randomly allocated to either the TA-65 (16 mg/day) or placebo group for a year. After this period, the TA-65 group exhibited a significant rise in the average total lymphocyte count, predominantly due to increases in CD3+, CD4+, CD8+T lymphocytes, B lymphocytes, and NK cells, relative to baseline. No lymphocyte increase was observed in the placebo group. After 12 months, a crucial inflammatory marker, high-sensitivity C-reactive protein, saw a 62% reduction in the TA-65 group compared to the placebo group, and the TA-65 group faced significantly fewer adverse events (97). Exploring the immunomodulatory properties of metformin on T cells, another study discovered that metformin elevates T cell telomerase activity, reduces the population of senescent CD8+ T cells, and curtails their SASP (98). Specifically, metformin suppresses IFN-γ secretion in aging CD8+ T cells and the production of the pro-inflammatory cytokine IL-6. While metformin exerts minimal influence on the secretion of granzyme B in aging T cells, it enhances TNF-α production. RNA-seq results indicated that metformin fosters the expression of genes linked to stemness and telomerase activity while repressing the expression of DNA damage-associated genes. This study posits that metformin holds promise as an agent for countering immune senescence and age-related diseases (98).

Additionally, TERT/telomerase activity in peripheral lymphocytes has been observed to be influenced by natural physiological factors, including diet and physical exercise. A recent randomized single-blind controlled crossover study involving twenty-two healthy participants assessed the impact of a short-term, five-day intervention using raw or cooked Brassica vegetable leaves. The intervention with both types of preparations led to a slight increase in telomerase activity in CD4+ cells. Notably, in CD8+ cells, telomerase activity significantly rose following the consumption of cooked vegetable samples. This enhancement in telomerase activity in T cells is believed to bolster cell-mediated immune responses (99). Furthermore, a meta-analysis examining the influence of both single sessions and long-term exercise training on TERT expression and telomerase activity revealed that exercise, regardless of duration, can elevate TERT and telomerase activity in non-cancerous cells of both humans and rodents. Endurance athletes, in comparison to their inactive counterparts, exhibited heightened TERT and telomerase activity in white blood cells, signifying that exercise training can positively modulate lymphocyte TERT expression and telomerase activity, conferring health benefits (100). These strategies for augmenting TERT expression and stimulating telomerase in lymphocytes are consolidated in **Table 1**.

**Table 1:** Strategies to Enhance TERT Expression and Telomerase Activation in Lymphocytes.

Cell type	Manipulation	Effects	References
T cell	Transfection with TERT gene	Preserves antigen-specific and holistic immune response in vivo, inhibits the growth of human melanoma xenografts in mice.	(90)
T cell	Transfection with TERT gene	Mitigates premature senescence and preserves immune reactivity of HIV-1 specific T cells.	(93)
NK cell	Transfection with TERT gene	Augments NK cell activation and proliferation, prolongs lifespan, enhances efficacy in NK cell-based cancer therapies.	(79)
T cell	Exposure to TAT2	Increases telomerase activity, decelerates telomere shortening, boosts proliferation potential, enhances functional cytokine and chemokine production to counteract HIV.	(94)
T cell	Exposure to atorvastatin	Boosts T cell proliferation, delays inflammatory response in arterial vessel walls, slows atherosclerosis progression.	(95)
NK, NKT-like and T cell	Exposure to IL-15	Promotes TERT upregulation, advantageous for adoptive cell therapies.	(96)
Lymphocytes	Exposure to TA-65	Elevates counts of CD3+, CD4+, CD8+ T lymphocytes, B lymphocytes, NK cells, reduces high-sensitivity C-reactive protein levels.	(97)
T cell	Exposure to metformin	Elevates T cell telomerase activity, diminishes CD8+ senescent T cell population, curtails their SASP.	(98)
T cell	Short-term (five days) intervention with Brassica vegetable leaves	Amplifies T cell telomerase activity, bolsters cell-mediated immune responses.	(99)
White blood cell	Physical exercise	Enhances TERT and telomerase activity in lymphocytes, confers health benefits.	(100)

In conclusion, the strategic enhancement of telomerase activity through genetic interventions, small-molecule manipulation, and dietary or physical exercise presents a viable method for improving lymphocyte functionality. The modulation of TERT and telomerase in lymphocytes is multi-faceted, with numerous transcription factor-binding sites within the TERT promoter suggesting its association with various cellular pathways. This association might uncover novel preventive targets for regulating TERT/telomerase expression and activity.

## 6 Future perspectives

This review encapsulates recent advancements in immunosenescence research, particularly focusing on T-cell senescence and the modulatory effects of TERT/telomerase. It is noteworthy that telomerase expression levels and enzymatic activity do not consistently correlate with telomere length or the senescence status of cells (101). Further investigation is imperative to thoroughly understand these intricate dynamics in human lymphocytes.

Earlier research indicated that the early regression of the thymus, responsible for producing naïve T cells, results in a post-puberty reduction in T cell production, diminishing the population of circulating naïve T cells. Subsequent studies, however, revealed that even in young adults, most T cells are derived from peripheral T cell proliferation rather than thymic output. The thymus’s contribution to T cell generation is estimated to decrease from about 16% to less than 1% in adulthood, a deficit potentially compensated by adjusted homeostatic proliferation (102, 103). Consequently, thymic involution is not the predominant factor in immunosenescence. Instead, the prolonged division of lymphocytes induced by long-term antigen exposure is identified as the primary cause of immunosenescence. It is also important to note that most data on immunosenescence are derived from mouse models, including those using genetically modified TERC/TERT knockout mice, partially mimicking the biological characteristics of human telomeres in various inflammatory responses (101). Due to the significantly shorter lifespan of mice compared to humans and their telomeres being 10 times longer than human counterparts, mouse telomere/telomerase characteristics do not fully replicate human cell behavior (104). Therefore, comprehensive research into telomere/telomerase dynamics in human lymphocyte during immunosenescence and inflammatory processes is crucial for confidently translating telomerase modification strategies into clinical treatments.

It is critical to acknowledge that upregulating TERT or activating telomerase might induce malignant cell transformation (85, 105), and such approaches should be avoided in therapeutic contexts. The design of small-molecule telomerase activators, like TAT2 and TA-65, targeting immune cells specifically, holds promise in preserving specific immune cell functions while minimizing potential adverse effects. Recent studies have highlighted that enhancing mitochondrial function and reducing oxidative stress through the overexpression of mitochondria-targeted TERT, delivered by adeno-associated virus serotype 9 with a TERT-encoding sequence fused to a mitochondrial targeting sequence, can improve cardiac function. This approach of targeting mitochondria avoids telomerase activation and the associated risk of malignant transformation due to TERT expression in the nucleus (106, 107). Moreover, future research should explore other regulatory strategies, such as stabilizing telomere caps and preserving cell-surface CD28, to potentially delay immunosenescence and treat age-related diseases.
